# Stem cell-derived cell sheet transplantation for heart tissue repair in myocardial infarction

**DOI:** 10.1186/s13287-019-1536-y

**Published:** 2020-01-08

**Authors:** Rui Guo, Masatoshi Morimatsu, Tian Feng, Feng Lan, Dehua Chang, Feng Wan, Yunpeng Ling

**Affiliations:** 1 Department of Cardiac Surgery, Peking University Third Hospital, Beijing, 100191 China; 2 Department of Cardiovascular Physiology, Graduate School of Medicine, Dentistry and Pharmaceutical Sciences,, Okayama University, Okayama, 700-8558 Japan; 3 Beijing Anzhen Hospital Beijing Institute of Heart Lung and Blood Vessel Disease Capital Medical University, Beijing, 10029 China; 4 Department of Neurology, Graduate School of Medicine, Dentistry and Pharmaceutical Sciences, Okayama University, 2-5-1 Shikatacho, Kitaku, Okayama, 700-8558 Japan; 5 Department of Cardiac Surgery, The University of Tokyo Hospital, 7-3-1 Honggo, Bunkyo-ku, Tokyo, 113-8655 Japan; 6 Department of Cardiovascular Surgery, Tongji University East Hospital, Shanghai, 200120 China

**Keywords:** Cell sheet, Cardiac tissue, Myocardial infarction, Heart failure, Cell therapy, Mesenchymal stem cells, Skeletal myoblasts, Pluripotent stem cells, Inflammation, Angiogenesis

## Abstract

Stem cell-derived sheet engineering has been developed as the next-generation treatment for myocardial infarction (MI) and offers attractive advantages in comparison with direct stem cell transplantation and scaffold tissue engineering. Furthermore, induced pluripotent stem cell-derived cell sheets have been indicated to possess higher potential for MI therapy than other stem cell-derived sheets because of their capacity to form vascularized networks for fabricating thickened human cardiac tissue and their long-term therapeutic effects after transplantation in MI. To date, stem cell sheet transplantation has exhibited a dramatic role in attenuating cardiac dysfunction and improving clinical manifestations of heart failure in MI. In this review, we retrospectively summarized the current applications and strategy of stem cell-derived cell sheet technology for heart tissue repair in MI.

## Introduction

In the process of animal development and evolution, the loss of mammalian cardiac regenerative potential may be due to the imbalance of thyroid hormones [1]. However, with the prolongation of human life, this loss of myocardial proliferative capacity has become the root cause of the serious consequences of various heart diseases [2]. After the loss of cardiomyocytes (CMs) caused by ischemia with insufficient myocardial oxygen supply, increased risks of morbidity, mortality, and disability form a severe burden on the daily life of patients with heart disease worldwide [3, 4]. The present therapeutic interventions, including traditional medicine, devices, and surgical therapies, have therapeutic effects on heart failure (HF); however, further revascularization and medical therapy may be useless because the ventricular remodeling process is usually irreversible in end-stage HF patients. Therefore, a new therapeutic strategy, such as regenerative medicine, is urgently needed to overcome these limitations [5].

Stem cell transplantation, as a new treatment strategy, has been reported to improve cardiac function in patients with advanced HF after MI [6–8]. Stem cell transplantation can enhance tissue perfusion, contribute to angiogenesis, and preserve or regenerate myocardial tissue, as proven by many basic research and clinical studies [5, 9–12]. The first application of stem cell transplantation to treat MI occurred in 2001 and showed encouraging results. Since then, more clinical studies have indicated that stem cells are safe and exhibit few treatment-related adverse events in comparison with control groups [5, 13]_._

**Fig. 1:**
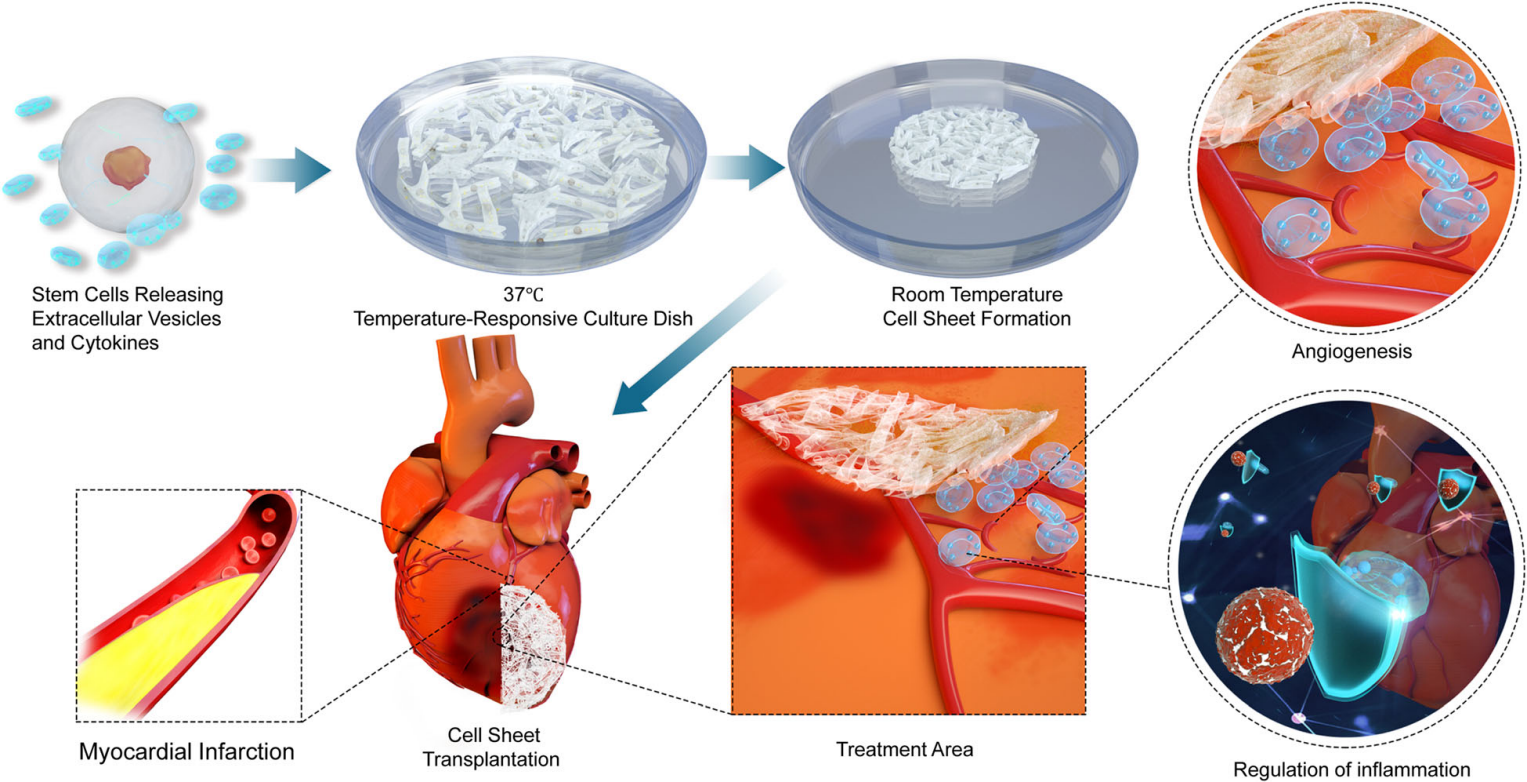
Cell sheet application and mechanism in ischemic heart disease

Several studies have shown that different types of stem cell transplantation facilitate graft survival and the formation of new blood vessels [5]. Recent research indicates acute immune response is the benefit of stem cell therapy in ischemic heart disease [14]. The cell sources used in cell transplantation for research associated with MI include autologous marrow mononuclear cells (MNCs), mesenchymal stem cells (MSCs), skeletal myoblasts (SMs), cardiac progenitor/stem cells (CPCs/CSCs), bone marrow mononuclear cells, human embryonic stem cell-derived cardiomyocytes (hESC-CMs), and human induced pluripotent stem cell-derived cardiomyocytes (hiPSC-CMs) [15, 16]. Although many basic studies and preclinical trials have been conducted over the past decade, the feasibility and effectiveness of these cells in acute MI and chronic heart diseases remain unclear. Among many factors influencing the outcome of myocardial cellular therapy, the transplantation approach is one of the most important. In the present review, we analyzed and discussed effective stem cell transplantation methods for myocardial repair in MI (Fig. 1).

In situ direct myocardium injection of cell suspension and intracoronary administration of cell suspension have commonly been used in acute MI [5, 16, 17]. Although the direct injection of cells can successfully mend small damaged areas, transplantation by the needle injection of a cell suspension easily causes aggregation and necrosis of the grafted cells, and the shape, size, and location of transplanted cells are difficult to control, which can lead to lower survival rates, marginal engraftment, and suboptimal outcomes [17–20]. Therefore, methods of cell transplantation are important to improve adhesion between the transplanted cells and the host heart tissue and thereby localize the cells on the heart tissue.

In recent years, bioscaffolds have been used for the regeneration of many tissues and organs. However, the clinical use of tissue engineering with scaffolds is extremely limited in CMs at present because of some defects. One is the challenge of firmly and compactly fixing CMs in scaffolds to form dense myocardial tissue. Another is the difficulty of embedding pervasive vascular networks through scaffolds, which means an important metabolic environment is lacking. A third is that dramatic inflammation, foreign body reaction, and arrhythmogenic potential commonly occur after long-term scaffold transplantation, undermining the therapeutic effects, problems that should be examined and completely addressed before clinical applications [18, 21, 22].

To address these obstacles, cell sheet, a scaffold-free technology, has been created using a temperature-responsive culture surface [23]. For this purpose, temperature-responsive culture dishes were developed by grafting with a polymer (poly-*N*-isopropylacrylamide) that exists in a hydrophobic state at 37 °C and a hydrophilic state below 32 °C. When the temperature is below 32 °C, the grafted polymer rapidly hydrates, causing it to expand and detach from the surface so that a viable monolayer cell sheet can be collected with full preservation of the cell-cell contacts and extracellular matrix. Compared with biodegradable scaffolds, scaffold-free cell sheets are nonenzymatic, intact, and connective structures of cultured cells obtained by a simple temperature change to ameliorate many problems related to the degradation of scaffolds [23].

Over the past 20 years, cell sheet technology has realized myocardial tissue regeneration in vitro by tissue engineering with increasing numbers of intact and connective cell-cell contacts [24–27]. Recent clinical studies indicated that cell sheet technology improved the ejection fraction, recovered the dysfunctional cardiac wall, increased vasculargenesis, and decreased fibrosis in heart disease models [28–32]. Even if several issues should be considered, including transplantation time window and lack of nutrition to cell sheet or hypoxia resulting in transplanted cell death prior to clinical applications, cell sheet still has great potential as a novel tissue engineering treatment for cardiac infarction. This review concentrates on current topics associated with stem cell-derived cell sheet or stem cell-derived CM cell sheet for heart tissue repair.

## Stem cell composition of cell sheet

The cell sources for sheet used in cardiac transplantation to damaged hearts with MI in animal models include fibroblasts [33, 34], endothelial cells [35], cardiomyocytes cocultured with endothelial cells [35], dermal fibroblasts cocultured with endothelial progenitor cells [33], SMs [36–41], mesenchymal stem cells from adipose tissue [42, 43], bone marrow [44, 45], menstrual blood [46], adipocytes [47], adipose-derived stem cells [42, 43], cardiomyocytes from neonatal animals [34], Sca-1 (+) CPCs and C-kit (+) CPCs from adult murine hearts [48, 49], and ESC/iPSC-derived pure cardiomyocytes [50] or multiple cardiac cell lineages composed of cardiomyocytes and vascular cells [51, 52]. Collectively, considering their benefits and further perspectives of clinical application, we reviewed research updates about the use of SM-, MSC-, and ESC/iPSC-CM-derived cell sheet in MI therapy in this current paper. SMs are the first cell source for the treatment of ischemic heart disease in the form of cell sheet [5]. Although there are some ethical issues regarding these cells and the mechanism of action is not clear, they provide a good basis for the application of other cells. MSCs have a long history of clinical application, including not only the treatment of ischemic heart disease but also the extensive clinical applications in other diseases [15, 16]. These applications confirm the safety and partial mechanism of action of MSCs. Despite the ethical issues of allogeneic transplantation, the best option for clinical treatment at this stage is still MSCs. Pluripotent stem cells (PSCs) mainly include two kinds of cells, ESCs and iPSCs [53]. ESCs are restricted on ethical grounds in some areas and therefore cannot be applied worldwide. With Yamanaka’s work on reprogramming adult cells to construct iPS cell lines, ethical issues are avoided while duplicating the benefits of ESCs [53]. The cells used to construct iPSCs include blood, skin fibrils, urothelium, and other adult somatic cells [53]. These cells can be reprogrammed in vitro to construct stable cell lines with strong proliferative capacity and directed differentiation function [53]. Although iPSCs require ethical review during allogeneic transplantation, the world’s leading research institutions are currently establishing iPSC cell banks for allogeneic transplantation, so it is expected that in the future, the clinical application of allogeneic transplantation will be subject to reduce ethical restrictions. Overall, SMs are the earliest cell source applied via the cell sheet technique to the clinical treatment of ischemic heart disease, which lays a solid foundation for future research. At present, MSCs are still the most suitable for clinical treatment. However, the most promising approach is the use of iPSCs and differentiated cells from iPSCs (Table 1).

**Table 1:** The summary of comparison of different stem cell-derived cell sheets in MI

Cell sources	Advantages	Disadvantages
Myoblast	The most extensive research	Limited potency of de novo cardiomyogenesis, arrhythmic risk
Mesenchymal stem cells	Low immunogenicity, no arrhythmia risk, extensive clinical safety experience, and stronger paracrine ability	Limited potency of de novo cardiomyogenesis
Induced pluripotent stem cell-derived cardiomyocytes	Strongest de novo cardiomyogenesis ability, convenient for standardized operation protocol, and potential for genetic modification	Trauma formation, limited vascularization, higher possibility of genetic and epigenetic mutations, and arrhythmic risk
Induced pluripotent stem cell-derived cardiomyocytes/vascular cells	Convenient for standardized operation protocol, potential for genetic modification, increased survival rate of cell sheets, long-term beneficial outcome, and vascularization	Existence of undifferentiated stem cells or other irrelevant cells and arrhythmic risk
Cell sheets with omentum flap or preconditioning	Increased survival rate of cell sheets, long-term beneficial outcome, and vascularization	Higher possibility of genetic and epigenetic mutations during preconditioning and side effects after application of omentum flap

### Skeletal myoblast-derived cell sheet

SMs are the subject of most of the research on cell sheet for the treatment of acute myocardial infarction (AMI). SMs have advantages including autologous transplantation, ischemia resistance, nonmyocyte lineage differentiation, and high proliferative potential [5]. In basic research, SMs have been applied to MI in various animal models, including rats, hamsters, dogs, and swine [36–41, 54–57]. All previous studies showed that SMs improved cardiac function with fibrosis suppression, increased systolic function, increased wall thickness, and enhanced neovascularization, indicating the sufficiency and feasibility of SMs for cell sheet-based therapy in clinical MI [28–32]. Moreover, Uchinaka and his colleagues reported that laminin α2-secreting fibroblasts enhanced the therapeutic effect of skeletal myoblast sheet via inhibiting the detachment of implanted myoblasts from the grafted myocardium [58]. Based on the data from Kainuma and his colleagues, skeletal myoblast cell sheet transplantation combined with transphrenic peritoneoscopy and omentopexy promoted arteriogenesis and improved coronary microcirculation physiology in a 6-week MI rat model [59]. The omentum is known to reduce inflammation and to promote revascularization, reconstruction, and tissue regeneration. Kainuma and his colleagues showed that myoblast-derived cell sheet therapy with omentum improves the hypoxic environment in a rat MI model, significantly enhancing the cell engraftment induced by the cell sheet [60]. In addition, they found a group of potentially relevant molecules, including VEGF-A, VEGF receptor-1, VEGF receptor-2, Akt-1, PDGFR-β, Ang-1, Tie-2, VE-cadherin, and PECAM, which were upregulated in the combined group compared with the cell sheet group, indicating enhanced paracrine action [60]. Additionally, Shudo et al. observed that better improvement in cardiac function, increased angiogenesis with increased expression of VEGF and STAT3 was induced by myoblast-derived cell sheet combined with omentopexy than by the cell sheet group alone in the porcine MI model [54]. Sekiya and his colleagues showed that mouse muscle-derived single-layer stem cell sheet supported pump function without cardiac arrhythmias in a chronic MI mouse model [61]. In addition to adult MI animal models, myoblast cell sheets also improved cardiac dysfunction via enhancing the endogenous regenerative abilities in an infant rat model of MI and exhibit a better therapeutic effect on infant hearts relative to adult hearts because infant hearts have a stronger cardiomyocyte proliferation capacity [62]. On the other hand, a clinical trial showed that autologous myoblast sheets have the potential for functional recovery in dilated cardiomyopathy and ischemic cardiomyopathy with or without left ventricular assist devices. However, additional larger-scale and long-term clinical trials should be conducted [29]. In addition to the beneficial characteristics of SM-derived sheet in MI treatment, we must clarify that the disadvantages of SM-derived sheet still exist. First, SM-derived sheets do not have the potency of de novo cardiomyogenesis, and SMs can cause arrhythmic events because they cannot form gap junctions with the host CMs due to the lack of expression of *N*-cadherin or connexin [41]. Moreover, it is suspected that fibrillation-like contraction and stretch-activated ion channel activation could be induced by SM-derived sheet [29]. Overall, we should consider improving the current SM-derived cell sheet by optimizing the composition of SM sheet or combining the use of synchronized contraction devices with SM sheet before application to clinical patients.

### Mesenchymal stem cell-derived cell sheet

Transplanted cell sheets based on mesenchymal stem cells from bone tissue, adipose tissue, and menstrual blood have demonstrated a certain extent of cardiomyogenesis and dramatical paracrine effects, which contribute to vascularization, cardioprotection, improved left ventricular function, and myocardial repair [15, 16]. Tanaka and his colleagues reported that autologous bone mesenchymal stem cell-derived cell sheet significantly improved left ventricular function with accelerated angiogenesis in the peri-infarcted area and decreased infarction volume and inhibited apoptosis at 4 weeks after transplantation in an 8-week rabbit MI model [45]. More importantly, they also showed that hypoxia preconditioning greatly strengthened the therapeutic effect of BM-MSC sheet [45]. Moreover, the same group also found that hypoxia-preconditioned mouse cardiosphere-derived cell sheet remarkably improved left ventricular function with reduced fibrosis and enhanced angiogenesis via the activation of the PI3-kinase/Akt signaling pathway in a 2-month mouse MI model [63], and human cardiosphere-derived cell sheet after hypoxic preconditioning enhanced cellular function via activation of the PI3K/Akt/mTOR/HIF-1α pathway in an in vitro study [64]. Adipose-derived stem cell (ADSC) transplantation has been thought to recover MI by direct injection [65]. With the technology of cell sheet, ADSCs and adipocyte-derived cell sheet have been constructed and used in MI research in animal models [66, 67]. Some studies showed that ADSC-derived cell sheet could attenuate cardiac dysfunction with scarred myocardium repair, cardiac remodeling attenuation, increased cellular engraftment, upregulated growth factor and cytokine expression, decreased interstitial fibrosis, and increased capillary density in border areas [66, 67]. Interestingly, an angiotensin II receptor blocker, irbesartan, was observed to abolish the effects of rat ADSC-derived cell sheet on the attenuation of cardiac dysfunction and remodeling in a 5-week AMI rat model [68]. Moreover, Imanishi and his colleagues reported that the transplantation of adipocyte-derived sheet 5 min after AMI surgery ameliorated inflammation and inhibited fibrosis in a mouse AMI model after 2 days or 28 days [47]. However, a recent study showed that human placental MSCs cannot differentiate into beating cardiomyocytes under cardiac differentiation medium treatment, but undifferentiated placental MSCs formed intact aligned cell sheet, giving rise to concern regarding the further utility of placental MSC-derived sheet in cardiac regeneration and repair [69]. Therefore, in future research, we should focus more on the combination of multiple cell sources in the fabrication of cell sheet. For example, some papers showed that the combination of fibroblasts expressing laminin G-module 4–5 of α2 and SMs in multilayered sheet markedly inhibited the detachment of implanted myoblasts from sheet, resulting in more permanent and stable efficacy on single myoblast sheet because fibroblasts could abundantly embed in the myoblast layer to fasten and support the structure via extracellular matrix production [58]. Another report showed that the local release of VEGF enhanced the transplantation efficiency of layered cardiomyocyte sheet, which could be related to vascularization [70]. Collectively, MSCs are a promising candidate for cell sheet applications, although some limitations should be addressed.

### Pluripotent stem cell-derived cell sheet

Although the reconstruction of myocardial tissue has been achieved by tissue engineering methods, the cell sources for myocardial tissue are still problematic. To date, many research teams have reported that fetal or neonatal cardiomyocyte-derived cell sheet could be used in cell therapy for MI in animal studies; however, the use of fetal or neonatal cardiomyocyte-derived cell sheet in humans presents a complex ethical dilemma, and the limited number of cardiomyocytes for clinical application through the above methods presents another major problem [15, 16]. Moreover, although somatic stem cells have been applied in producing cell sheet for heart repair in animal models because of their cardiomyocyte differentiation potency and their paracrine action, their differentiation ability has always been controversial and widely considered too limited to produce enough functioning cardiomyocytes for clinical application, and the MI repair effect of their paracrine action should still be examined in animal models. Fortunately, pluripotent stem cells with the property of infinite proliferation and highly efficient cardiomyocyte differentiation, including ESCs and iPSCs, are highly advantageous as cell sources for cell sheet for use in MI repair. Cardiac cell sheets using ESC-derived cardiomyocytes have been developed. A cell sheet composed of CPC derived from ten Rhesus monkey ESCs generated new cardiomyocytes and trophic support at 8 weeks after transplantation in a 10-week monkey MI model [71]. Matsumoto and his colleagues fabricated a self-pulsating cell sheet composed of CMs, endothelial cells, and Mural cells differentiated from ESCs, named “cardiac tissue sheet (CTSs),” and demonstrated that the three-layered CTSs attenuated cardiac dysfunction at 1 week after transplantation in a 2-week rat AMI model partly due to neovascularization mediated by indirect paracrine effects. However, the engraftment efficiency of mouse ESC-derived CTSs was rather low at 4 weeks after transplantation, which indicates that it is necessary to investigate another cell source for better cell survival and myocardial regeneration in further studies [51]. Compared with ESCs, iPSCs can be generated from patients’ own somatic cells without ethical problems for personalized therapy. iPSC-differentiated cell sheets have been investigated for the treatment of MI [15, 16]. Early in 2012, Miki, Sawa, and their colleagues first developed CTSs from mouse iPSCs and confirmed that these highly pure mouse iPSC-derived CM sheet survived and alleviated left ventricular remodeling at 4 weeks after transplantation in a 6-week rat MI model [72]. Meanwhile, highly pure (almost 90%) human iPSC-derived CM sheet were created by Kawamura, Sawa, and their colleagues from Osaka University [50]. They first showed that human iPSC-differentiated highly pure CM sheet could improve cardiac function through attenuating left ventricular remodeling, increasing neovascularization mainly due to paracrine action, inhibiting fibrosis, and inducing cardiomyogenesis at 8 weeks after transplantation in a 12-week MI porcine model [50]. In 2013, the same group found that the omental flap enhanced the survival rate of human iPSC-CM-derived cell sheet in a porcine heart at 1, 4, and 8 weeks via increased angiogenesis compared with previous single iPSC-CM-derived cell sheet [73]. Next, in 2017, they showed that the omental flap significantly enhanced the survival of transplanted human iPSC-derived CM sheet and the therapeutic effects of iPSC-CM-derived cell sheet in an MI porcine model, indicating that this system could be used to treat severe HF [74].

Moreover, Matsumoto and his colleagues transplanted CTS from human iPSC-derived CMs consisting of 67–85% CMs, 8–13% endothelial cells, and 3–19% Mural cells into a rat MI model and showed a functional recovery as long as 2 months after transplantation [75]. Functional and electrical recovery with cyclic contraction of transplanted cells was observed 2 weeks after the transplantation of mouse iPSC-CM-derived cell sheet in a 4-week MI rat heart [75]. A research group successfully used the fibrin gel-enhanced delivery of human iPSC-CM sheet to decrease cardiac infarction at 4 weeks after transplantation via vascularity, to decrease apoptosis, and to increase engraftment rates in a mouse model at 4 weeks of MI [76]. Recently, clinical-sized large CTSs composed of 32.4–58.8% CMs, 0.9–9.7% endothelial cells, and 6.3–35.1% MCs were created by Ishigami and his colleagues [77]. They confirmed that L-CTS transplantation attenuated left ventricular remodeling and improved cardiac dysfunction with higher systolic function of the left ventricular, higher ejection fraction of the left ventricular, increased circumference strain in infarct border regions, lower fibrotic area, and higher capillary density in the border region at 4 weeks after transplantation in a 6-week AMI porcine model [77]. Considering the substantial advantages of unlimited proliferation ability and directional differentiation function, iPSC-CMs could be the most feasible cell source for myocardial cell therapy for clinical MI. In the PSC differentiation study, the earliest embryonic body differentiation protocol obtained inefficient differentiation of cardiomyocytes [78] but provided an important research basis for the differentiation function of PSCs. Thus, the differentiation efficiency was improved, a differentiation scheme using a protein-induced monolayer culture such as Activin A and BMP4 [79] was developed, and the efficiency of classification into cardiomyocytes was improved by using hiPSCs. However, the differentiation efficiency of this method still varies considerably between batches. With the development of the chemically defined [80] method, the efficiency of the differentiation of hiPSCs into CMs is guaranteed by the use of a serum-free system combined with chemical small molecule induction. With the development of bioreactor culture technology [81], an automated scheme for the cultivation and directed differentiation of hiPSCs was found, making it possible to produce hiPSC-CMs in large amounts. The main reason for limiting the use of hiPSCs for large-scale clinical treatment is the safety issue. Since the reprogramming transcription factor of hiPSCs contains the oncogene c-Myc, hiPSCs themselves have a certain tumorigenicity. Moreover, the electrical activity of hiPSC-CMs caused by insufficient differentiation maturity is inconsistent with the frequency of electrical activity of myocardial cells in patients, which may lead to malignant arrhythmia, which is another key problem faced by hiPSC-CMs. Therefore, before the application of hiPSCs and their differentiated cells to clinical treatment, further experiments are needed to address the above problems. Overall, hiPSC-CMs could be a potential cell source for the future application of tissue bioengineering sheet in clinical patients with MI.

Collectively, based on our expertise, we think that human iPSC-derived multiple layered cell sheet with special materials such as gelatin hydrogel microspheres or collagen-based vascular beds undergoing preconditioning before transplantation will be the future trend in the development of cell sheet for MI treatment because this approach has the strongest ability to generate large-scale cell sheet with good vascularization and long-term survival and without ethical and immunological issues, which indicates broad clinical application potential in MI patients.

## Cell sheet-based cardiac engineering

Although cell sheet techniques have many advantages over conventional tissue engineering methods, some problems still should be addressed. A single cell sheet is too thin to support long-term beneficial effects without vascularization in the hypoxic pathological condition of MI. To overcome such critical issues, cell sheets are often overlapped. However, necrosis and a decreased survival rate can occur in multilayered cell sheet because of hypoxia or limited nutrition. Therefore, we must consider a 3D-layered cardiac graft with vascularization obtained in the manner of myocardial tissue engineering by layering cell sheet.

Extracellular matrices and adhesion proteins can be adhered to detached cell sheet, providing the possibility of fast establishment of tissue by layering cell sheet [82]. Between two sheets, the gap junction is firmly established partially via connexin 43, allowing electrical communication to be established within 30 min and two sheets to exhibit spontaneous beating after the layering, indicating that three-dimensional cardiac tissue could be fabricated by layering monolayered sheet [82]. Furthermore, a study showed that there is a bridge crossing the barrier between the cell sheet and the host, which could enable realistic cardiac function because of the synchronized beating [83]. Overall, due to the preserved extracellular matrix proteins, multiple cell sheets can be stacked tightly by simple layering with synchronization. Additionally, gap junctions were observed between cell sheet and the host, and small molecules provided communication by passing through functional gap functions [34]. Based on previous studies, 80 μm is the thickest layered cell sheet used in subcutaneous tissue in a single transplantation. However, to prolong the survival time of cardiomyocytes, fabricate more functioning grafts, and achieve better therapeutic effects in chronic HF, it is critical to develop thicker vascularized tissue engineering sheet [84]. To produce large-scale cell sheet constructs, vascularization is required to provide sufficient oxygen and nutrients within such thick tissues. To accelerate vascularization within engineered tissues, coculture with endothelial cells is a promising approach. Endothelial cells can be incorporated among cell sheet and promote vascular networks and connections to the host vasculature after transplantation [85]. To further maintain the long-term survival of thick 3D tissues, a technique for producing mature blood vessels is necessary. A vascular bed consisting of a resected section of femoral tissue with a connectable artery and vein is applied to cell culture with layered cardiomyocyte sheet and endothelial cells [85]. In a bioreactor system, new functional and mature vessels can connect to the vasculature in the vascular bed, which is continuously perfused with culture medium [85]. A collagen-based vascular bed containing microchannels has also been applied for vascular network formation. This important culture system dramatically induced the differentiation of iPS cells into cardiomyocytes [85]. Therefore, in vitro cell-dense tissues with the desired thickness can be fabricated by multiple cell sheet layering. Additionally, Matsuo et al. inserted gelatin hydrogel microspheres (GHMs) between each cardiovascular cell sheet to break the viability limitation via appropriate spacing and fluid impregnation with GHMs [86]. Fifteen sheets with GHMs (> 1 mm thickness) were stacked within several hours and viable after 1 week in vitro. Using GHMs, large viable 3D cardiac structures from pluripotent stem cells can be generated within several hours [86]. Compared with 1 month for tissue generation by the traditional method, this method is easier and more efficient. They also demonstrated that mouse ESC-CM-derived sheet with biomaterials successfully survived a long time and recovered AMI in a rat AMI model with high engraftment efficiency, original microcapillary network between host and graft at 4 weeks after transplantation, and sufficient perfusion of the whole regenerated myocardium at 3 months after transplantation [86, 87].

Tissue engineering based on cell sheet has been shown to promote tissue modeling in regenerative medicine [15]. Stem cell-derived cell sheet-based cardiac tissue engineering has already shown more beneficial effects in MI than direct injection or the administration of stem cells with 3D scaffolds [15]. To better construct functional 3D cell sheet-based cardiac tissues, multiple-layer (up to 12 layers) cardiac cell sheet can be produced on a vascular bed to incorporate different types of cell sheet. However, the extended time required to prepare many sheets and to layer sheet is still a problem [85]. Recently, the utility of GHMs has dramatically reduced the time cost, illustrating the progress researchers have made in developing cell sheet technology [86, 87]. Moreover, synthetic substrates, such as polylactic acid, polyglycolic acid, and copolymers, have great promise for addressing additional challenges presented in the storage, transport, and clinical delivery of cell sheet in the field of cardiac tissue engineering [88].

## Function of cell sheet in MI

Cell sheet can ameliorate MI in both the acute phase and the chronic phase of MI with observably enhanced systolic function, increased cardiac wall thickness, dramatic neovascularization, decreased cell graft apoptosis, and inhibited cell graft inflammation in animal models with less than 3 months of MI, especially small animal models (Table 2). Regarding the clinical application of cell sheet, only limited studies have shown the partial function of myoblast-derived cell sheet in patients with ischemic cardiomyopathy and dilated cardiomyopathy [28–32]. Before effective large-scale clinical trials, we must provide more convincing proofs of the marked function of cell sheet in large animal models in a long-term AMI. Furthermore, we should also improve our understanding of the cellular and molecular mechanisms underlying the improvement of cardiac function after cell sheet transplantation.

**Table 2:** The summary of stem cell-derived cell sheet transplantation experiments in MI

Cell sources	Model	Species	Cardiac function	Wall thickness	Neovasculization	Fibrosis	Trophic factors	Reference
Myoblasts (rat)	MI	Rat	Improvement of EF	Increased	Not mentioned	Decreased	SDF-1, HGF, VEGF	36
Myoblasts (rat)	MI	Rat	Improvement of EF	Increased	Increased	Decreased	SDF-1, HGF,VEGF	37
Myoblasts (porcine)	MI	Porcine	Improvement of EF	Not mentioned	Increased	Decreased	No difference	40
Myoblasts (rat)	MI	Rat	Improvement of EF	Increased	Increased	Decreased	Not mentioned	55
Myoblasts + Bcl-2 (rat)	MI	Rat	Improvement of EF	No difference	Increased	Decreased	VEGF, PLGF	56
Myoblasts + Bcl-2 (rat)	MI	Rat	Improvement of EF	Increased	Increased	Not mentioned	Not mentioned	57
Myoblasts + OP (porcine)	MI	Porcine	Improvement of EF	Not mentioned	Increased	Decreased	VEGF, STAT3	54
Myoblasts + HGF (rat)	MI	Rat	Improvement of EF	No difference	Increased	Not mentioned	No difference	92
Myoblasts + laminin alpha2-secreting fibroblasts (rat)	MI	Rat	Improvement of EF	Increased	Increased	Decreased	HGF, IGF-1, VEGF	58
Myoblasts (rat)	MI	Infant rat	Improvement of EF	Increased	Increased	Decreased	Not mentioned	40
Myoblasts (porcine) + TPP with an OP	MI	Porcine	Improvement of EF	Not mentioned	Increased	Not mentioned	Not mentioned	59
Mesenchymal stem cells (rat adipose tissue)	MI	Rat	Improvement of EF, inhibition of LV dilatation	Increased	Increased	Not mentioned	HGF, VEGF	42
Mesenchymal stem cells (rat bone marrow)	MI	Rat	Improvement of EF	Not mentioned	Increased	Decreased	Not mentioned	44
Mesenchymal stem cells (human adipose tissue)	MI	Rat	Improvement of EF	Not mentioned	Not mentioned	Not mentioned	Not mentioned	43
Mesenchymal stem cells (rabbit bone tissue) + hypoxic preconditioning	MI	Rabbit	Improvement of EF	Not mentioned	Increased	Not mentioned	VEGF	45
Mesenchymal stem cells (human menstrual blood)	MI	Rat	Improvement of EF, inhibition of LV dilatation	Not mentioned	Not mentioned	Decreased	No difference	46
Adipocyte (mouse adipose tissue)	MI	Mouse	Improvement of EF	Not mentioned	Not mentioned	Decreased	Adiponectin	47
ADSC (rat adipose tissue)	MI	Rat	Improvement of EF	Not mentioned	Increased	Not mentioned	Not mentioned	67
ADSC (mouse adipose tissue)	MI	Rat	Improvement of EF	Increased	Increased	Decreased	Adiponectin, SDF-1, VEGF, IGF-1, bFGF, collagen I, laminin, fibronectin, vimentin	66
Endothelial progenitor cells (rat peripheral blood)	MI	Rat	Improvement of EF	Not mentioned	Increased	Decreased	No difference	33
Sca-1(+) cardiac progenitor cells (mouse)	MI	Mouse	Improvement of EF, inhibition of LV dilatation	Not mentioned	Increased	Decreased	VCAM-1	48
Muscle-derived stem cells (mouse)	MI	Mouse	Improvement of EF, inhibition of LV dilatation	Increased	Increased	Decreased	VEGF	61
Cardiaomyocytes/vascular cells (mouse ES cells)	MI	Mouse	Improvement of EF, inhibition of LV dilatation	Increased	Increased	Not mentioned	VEGF	51
Cardiomyocytes/vascular cells + GHMs (mouse ES cells)	MI	Mouse	Improvement of EF	Not mentioned	Increased	Not mentioned	Not mentioned	86
Cardiomyocytes (human iPS cells)	MI	Porcine	Improvement of EF, inhibition of LV dilatation	Increased	Increased	Decreased	VEGF, bFGF	50
Cardiomyocytes (mouse iPS cells)	MI	rat	Improvement of EF	Increased	Increased	Decreased	Not mentioned	72
Cardiomyocytes (human iPS cells) + OF	MI	Porcine	Improvement of EF	Not mentioned	Increased	Not mentioned	VEGF, SDF-1, bFGF	73
Cardiomyocytes/vascular cells (human iPS cells)	MI	Rat	Improvement of EF	Increased	Increased	Decreased	Not mentioned	75
Cardiomyocytes (human iPS cells)	MI	Mouse	Improvement of EF	Not mentioned	Increased	Not mentioned	Not mentioned	76
Cardiomyocytes/vascular cells (human iPS cells)	MI	Porcine	Improvement of EF	Increased	Increased	Decreased	Not mentioned	77
Cardiomyocytes (human iPS cells) + OF	MI	Porcine	Improvement of EF	Not mentioned	Increased	Not mentioned	VEGF, SDF-1, bFGF	74

*MI* myocardial infarction, *OP* omentopexy, *HGF* hepatocyte growth factor, *TPP* transphrenic peritoneoscopy, *ADSC* adipose-derived stromal cell, *ES* embryonic stem, *GHMs* gelatin hydrogel microspheres, *iPS* induced pluripotent stem, *OF* omental flap, *EF* ejection fraction, *LV* left ventricle

### Myocardial function

It is essential to improve cardiac function in terms of contraction. Cell sheet derived from most cell sources can contract synchronously in hearts, but the exception is SMs [18]. Although SMs cannot contract synchronously with the host myocardium and do not have electromechanical coupling with the host myocardium, some data have shown that skeletal myoblast sheets recover diastolic and systolic function in the infarcted region of hearts without contraction of the transplanted myoblasts partly due to extensive angiogenesis [89, 90]. Based on previous reviews, stem cell-derived cell sheet greatly improved systolic function and somewhat improved diastolic function in the chronic phase of MI animal models, and moreover, neovascularization and paracrine effects also contribute markedly to cardiac function recovery [15, 16, 18, 29].

### Paracrine effects and neovascularization

Many results have indicated that stem cell-derived cell sheets improve cardiac function in MI mainly via paracrine action. Implanted cell sheet can induce host tissues to neovascularize via secreting cytokines [36, 37, 39]. MSCs are considered to have an important role in effective paracrine action [90]. Marrow stem cell-derived cell sheet can strengthen angiogenesis via activation of the PI3-kinase/Akt signaling pathway in AMI animal models [63]. Hypoxia, as a major stress-inducing angiogenesis, can enhance the therapeutic effects of cell sheet in MI via increased vascularization [45]. An angiotensin II receptor blocker, irbesartan, observably abolished the effects of rat ADSC-derived cell sheet on the attenuation of cardiac dysfunction and remodeling in a 5-week MI rat model [68]. It is known that SMs do not easily develop synchronized beating with the host heart; however, studies have shown that improved systolic and diastolic functions exist in the host AMI heart after myoblast transplantation, even without contraction of the transplanted myoblasts, and observable angiogenesis was detected in the transplanted regions, indicating a plausible reason for the role of cell sheet in heart contraction recovery after MI [89, 90]. Moreover, a combination of c-kit-positive CPCs and endothelial progenitor cell-derived cell sheet improved the function of endocardial scar tissue more effectively than single CPC-derived cell sheet [91]. Considering the poor cardiomyocyte transdifferentiation capacity of c-kit-positive CPCs in this study, these data may be another proof of the role of neovascularization induced by cytokines from paracrine action in cardiac function recovery [91]. On the other hand, although some studies suggested that pure CM sheets derived from pluripotent stem cells possess functional recovery with synchronous contraction in small animal models of AMI, pluripotent stem cell-derived mixed cell sheets containing CMs, endothelial cells, and Mural cells could have better effects on the recovery of cardiac function due to their richer vasculature network and longer retention of cardiomyocytes in MI animal models, especially in the chronic phase [50–52, 71–73]. Matsumoto and colleagues found that few remaining CMs and many newly formed vessels stimulated by cytokines from the host existed in a rat AMI model [51]. More importantly, increasing data demonstrated that a combination of CM sheet and cytokines or omentum is a more effective approach to promote angiogenesis and may be more attractive for the treatment of AMI [72, 92].

### Regulation of inflammation

In the inflammatory response in the acute phase after MI, dying CMs and neutrophils secrete many cytokines/chemokines, including interleukin-1 and tumor necrosis factor-α, which can markedly induce cell death in ischemic area [93]. Therefore, the acute phase could be pivotal timing for cell sheet transplantation. The subsequent phase is the proliferation and healing process, which involves the secretion of many proliferative or prosurvival cell factors, including transforming growth factor-β and interleukin-10, which promote ventricular remodeling. Several data have shown that inflammatory modulation is crucial for the recovery of cardiac function after the cell sheet transplantation in MI, and cell sheet can attenuate the inflammation [94] and enhance the expression levels of anti-inflammatory-related genes [95]. However, recent research suggests that the functional benefit of stem cell therapy is from an acute inflammatory-based wound healing response [14]. Consequently, the effect of cell sheet on the inflammation of the host cardiac cells should receive attention in further studies.

## Further considerations for cell sheet therapy in MI

### Arrhythmias

Fatal arrhythmias are a major reason to limit the development of SMs as a therapeutic approach to MI, not only in terms of direct injection but also in terms of cell sheet. Based on current medical advances, an implantable cardioverter defibrillator could be used to prevent sudden cardiac death due to fatal arrhythmias induced by SMs. However, safety, feasibility, and cost should be further considered and studied. It remains unclear whether MSCs or pluripotent stem cells induce arrhythmia. However, many reports have shown that pluripotent stem cell-derived CMs or CM layered sheets have gap junctions with connexin 43 and beat synchronously with host hearts [18]. These data support the safety of pluripotent stem cell-derived CM layered sheet in MI therapy. However, the complications between native 3D thick myocardium and layered cell sheet should still be carefully investigated and monitored in further studies before clinical application.

### Immunological rejection

Immunological rejection is nearly nonexistent in studies associated with stem cell-derived cell sheet in the therapy of MI animal models because stem cells from animal models are syngeneic. The establishment of patient-derived stem cells usually requires more time and cost, and the quality cannot always be reliable for cell therapy. Therefore, stocked stem cells from other healthy donors with homozygous human leucocyte antigen are used in iPSC-derived CM sheet. We must be aware that the safety of such cell sheet should be carefully checked before clinical application. Tano and his colleagues reported that allogeneic rat mesenchymal stromal cells improved cardiac function to an equivalent degree compared to syngeneic MSC sheet despite an immunologic response without immunosuppressive drugs [96].

hiPSCs, if obtained by patient autologous cell reprogramming, require a significant amount of time and money, and consistent quality cannot be guaranteed between different patients. Based on the unlimited proliferation ability of hiPSCs, the best method is to select stable hiPSC cell lines for allogeneic transplantation. It is urgent to solve the problem of immune rejection caused by allogenic transplantation before application to clinical treatment. Deuse [97] and his team established a nonimmunogenic iPSC line with the major histocompatibility complex (MHC) class I and II genes inactivated and CD47 overexpressed. Mttapally et al. [98] knocked out the B2M and CIITA genes, which are crucial for the display of human leukocyte antigen (HLA) class I and II proteins at the cellular surface, to provide a source of universal donor iPSCs for the treatment. In summary, by knocking out the relevant genes for MHC I/II and HLA I/II, it is possible to construct cell lines that are more suitable for transplantation. These studies will help make hiPSCs safer for clinical treatment.

### Limited blood perfusion and biomaterial-supported engineered cardiac tissue

Limited blood perfusion is another problem for transplanted cell sheet, especially thick layered cell sheet [18, 87]. To solve this problem, a vasculature network of cell sheet must be established among cells in the sheet or between the cell sheet and host heart to achieve better and longer effects on cardiac function, as approaches include the combination of myoblasts and angiogenic factors or other types of cells [51, 92], the combination of cell sheet with omentum [73, 74], the use of layered stem cell-derived CTSs including multiple cardiac cell lineages [72, 75], and the application of GHMs or bioreactors with cell sheet [99, 100]. The purpose of these modifications is to generate thick stacked cell sheet with a functional vascular network. Recently, another technology to generate 3D engineered cardiac tissues was invented by using iPSC-derived cardiac cells and biomaterials [87, 101]. Some data showed that engineered cardiac tissues induced the formation of a vascular network originating from both host and engineered cardiac tissues [86]. More importantly, such a vascular network functioned to perfuse the regenerated myocardium at 4 weeks after transplantation in a rat MI model, survived between the host and graft for a long time, and improved cardiac dysfunction [86]. From single-layer cell sheet to layered cell sheet and 3D engineered cardiac tissues, stem cell-based cardiac regenerative therapy is increasingly effective. Further engineered 3D heart tissue or more effective methods to generate engineered cardiac tissues for better therapeutic effects on MI are expected.

## Conclusion

In this review, we have retrospectively discussed many essential achievements associated with stem cell sheet technology for cardiac repair. With the progress of stem cell sheet technology, increasing stem cell-derived sheets have already been studied in basic research and clinical trials. We have learned that stem cells are a feasible and promising cell source for cell sheet fabrication and that stem cell sheet transplantation can generate improved outcomes in MI therapy. It remains, however, to clarify the mechanisms that are involved in the therapeutic effects of stem cell sheet on cardiac dysfunction. Additionally, more advanced strategies to induce vascularization between 3D engineered cardiac sheet or tissues and host hearts in order to accelerate the development of stem cell-based cardiac regenerative therapy are still worth investigating in basic research and clinical trials. We believe that the promising applications of stem cell-derived cell sheet therapy in MI will be increasingly attracted in the next decade.

## Data Availability

Not applicable

## Abbreviations

ADSCs: Adipose-derived stem cells
AMI: Acute myocardial infarction
CMs: Cardiomyocytes
CPCs/CSCs: Cardiac progenitor/stem cells
CTSs: Cardiac tissue sheets
GHMs: Gelatin hydrogel microspheres
hESC-CMs: Human embryonic stem cell-derived cardiomyocytes
HF: Heart failure
hiPSC-CMs: Human induced pluripotent stem cell-derived cardiomyocytes
HLA: Human leukocyte antigen
MHC: Major histocompatibility complex
MI: Myocardial infarction
MSCs: Mesenchymal stem cells
PSCs: Pluripotent stem cells
SMs: Skeletal myoblasts
